# Computational modeling of chromatin accessibility identified important epigenomic regulators

**DOI:** 10.1186/s12864-021-08234-5

**Published:** 2022-01-08

**Authors:** Yanding Zhao, Yadong Dong, Wei Hong, Chongming Jiang, Kevin Yao, Chao Cheng

**Affiliations:** 1 Department of Medicine, Baylor College of Medicine, Room ICTR 100D, One Baylor Plaza, Baylor College of Medicine, Houston, TX 77030 USA; 2 The Institute for Clinical and Translational Research, Baylor College of Medicine, Room ICTR 100D, One Baylor Plaza, Baylor College of Medicine, Houston, TX 77030 USA; 3 Department of Electrical and Computer Engineering, Texas A&M University, College Station, TX 77843 USA

**Keywords:** ENCODE, Chromatin accessibility, Histone modifications, Transcription factor, Machine learning

## Abstract

Chromatin accessibility is essential for transcriptional activation of genomic regions. It is well established that transcription factors (TFs) and histone modifications (HMs) play critical roles in chromatin accessibility regulation. However, there is a lack of studies that quantify these relationships. Here we constructed a two-layer model to predict chromatin accessibility by integrating DNA sequence, TF binding, and HM signals. By applying the model to two human cell lines (GM12878 and HepG2), we found that DNA sequences had limited power for accessibility prediction, while both TF binding and HM signals predicted chromatin accessibility with high accuracy. According to the HM model, HM features determined chromatin accessibility in a cell line shared manner, with the prediction power attributing to five core HM types. Results from the TF model indicated that chromatin accessibility was determined by a subset of informative TFs including both cell line-specific and generic TFs. The combined model of both TF and HM signals did not further improve the prediction accuracy, indicating that they provide redundant information in terms of chromatin accessibility prediction. The TFs and HM models can also distinguish the chromatin accessibility of proximal versus distal transcription start sites with high accuracy.

**Supplementary Information:**

The online version contains supplementary material available at 10.1186/s12864-021-08234-5.

## Introduction

Chromatin accessibility is the extent to which nuclear molecules, including TFs, chromatin remodelers and histones, could physically interact with chromatinized DNA [1]. During the development, timely opening of specific genomic regions is essential for transcription activation and maintaining the regular differentiation state in cells [2, 3]. The chromatin remodeling either happens at the promoter locus to directly initiate the gene expression, or in the regulatory regions such as enhancer and silencer to impact the gene expression [4, 5]. To measure the opening genomic regions in cells, many sequencing-based techniques have been developed, with ATAC-seq now being the most widely used technique [6, 7]. Histone modifications (HMs) and transcription factors (TFs) are important determinants in chromatin accessibility [1, 8–10]. HMs regulate the chromatin accessibility by modulating the nucleosome affinity for active chromatin remodelers or changing the local chromatin structure [1, 11]. The combination of HMs, known as histone code, could dramatically change the state of the chromatin, allowing for the bindings of TFs or other active chromatin remodelers [12]. On the other hand, TFs dynamically compete with histones or interact with other chromatin binding proteins to promote the access to DNA [1]. According to the Encyclopedia of DNA elements (ENCODE), TFs play important roles in regulating chromatin accessibility as more than 90% of the accessible genome were bound by TFs [13]. In addition to TFs and HMs, other factors, such as the sequence composition or the enrichment of TF binding motifs, were also found to be associated with chromatin accessibility [14]. Though all associated with chromatin accessibility, there is a lack of quantitative, systematic studies that evaluate the different contributions of each factor during chromatin remodeling.

Many methods have been developed to predict the chromatin accessibility using genomic and epigenomic features. However, most of them only used DNA sequence information as the feature for prediction, leaving the contribution of other genomic and epigenomic features being un-answered [15–18]. For the limited studies that integrated DNA sequence with TFs for prediction [14], the different contributions between these factors in chromatin accessibility prediction was not examined. In addition, though over 20 HMs have been identified to be associated with chromatin accessibility [11, 19], which HM functions as the most important regulator remains unknown. It’s also unclear if HMs perform similarly with TFs or DNA sequence in regulating chromatin accessibility and if HMs and TFs have similar patterns in regulating chromatin accessibility among different cell lines. Therefore, a quantitative chromatin accessibility prediction model that integrates different epigenomic and genomic features is needed.

In order to build an integrative chromatin accessibility prediction model, we used the ChIP-seq data of 11 HMs and over 100 TFs from GM12878 and HepG2 cell lines. These datasets included TF binding profiles, HM patterns across the whole genome, DNA sequence information and 687 TF binding motif counts in accessible and non-accessible regions. The prediction performance of those 4 features was systematically studied and we found the HMs and TFs were the major regulators. Further applying the model revealed that different cell lines shared similar HMs that account for chromatin accessibility, yet only a few common TFs were identified between different cell lines that could predict the chromatin accessibility. In summary, we developed a computational framework for modeling the association between chromatin accessibility and different epigenomic and genomic features. This framework and the models introduced in this work could be applied to different datasets or species to study the regulation of chromatin accessibility.

## Results

### The integrative model for predicting chromatin accessibility

**Fig. 1:**
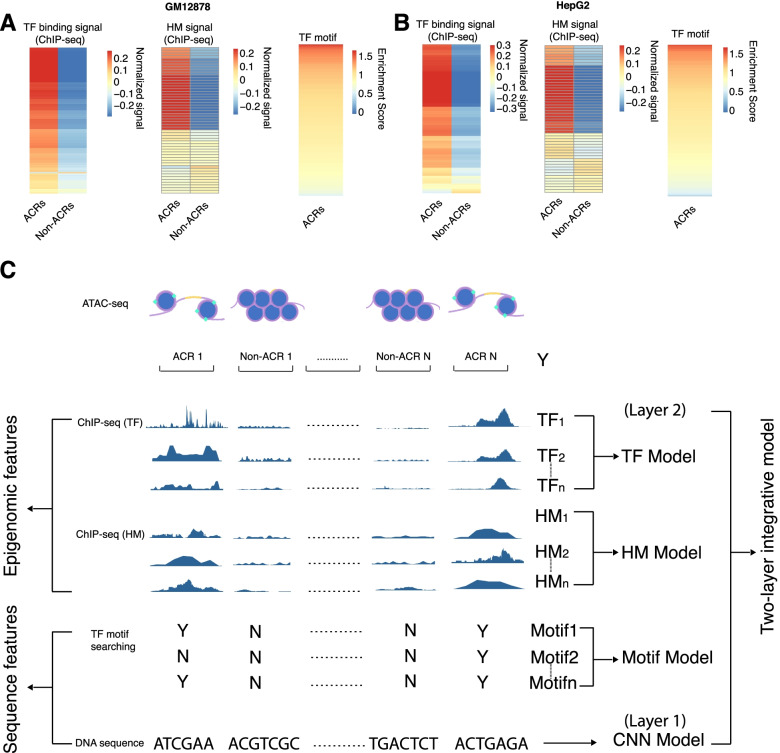
Integrative model configuration. **A** and **B**. Heatmap showing the signal difference of TFs and HMs in accessible and non-accessible regions and the enrichment of TF motifs in 895,695 accessible regions in GM12878 **(A)** and 883,018 accessible regions in HepG2 **(B)**. The signal was calculated by taking the average of all TFs/HMs binding signals. **C**. The chromatin accessibility of GM12878 and HepG2 data were acquired from ENCODE database. The DNA-sequence in the accessible region was extracted and embedded into the CNN model to convert the DNA sequence information to the probability of accessibility (Layer 1 model). The resulting probability was next integrated with ChIP-seq signal of HMs and TFs and the TFs motif occurrence in the accessible regions to train a Random Forest model

Our hypothesis was that the sequence, TF motifs, TF binding, and HMs were associated with chromatin accessibility. We validated the hypothesis by examining the signal difference between accessible and non-accessible regions. In both GM12878 and HepG2 cell lines, most of the TFs and HMs presented distinctive patterns between accessible and non-accessible regions. The binding signals for most TFs were enriched in the accessible regions while HM signals were enriched in either accessible or non-accessible regions (Fig. 1A-B). In addition, the enrichment score of TFs motifs validated that TF signals were associated with accessible chromatin regions. Compared to the background, most TFs motifs had enrichment score higher than 1, indicating the association between TF motifs and chromatin accessibility (Fig. 1A-B).

We then developed a two-layer integrative model to integrate different types of features for predicting chromatin accessibility (Fig. 1C). The chromatin accessibility measured by ATAC-seq was used as the training reference, and the peak region was further binned using 100 bp as the window size so that each of the open region had the same length. In the first layer, we used the DNA sequence in the accessible and non-accessible region to train a Convolutional Neural Network (CNN) model. Taking advantage of the CNN model, we converted any given DNA region into the probability of accessibility for sequence feature extraction. In the second layer, we integrated the derived probability of accessibility from the CNN model (first layer) with TF motif counts, HM, and TF binding signals for chromatin accessibility prediction. Specifically, TF motif counts were calculated based on the occurrences of TF motifs in the accessible and non-accessible region. HM and TF binding signals were derived from the bigwig files, which contained the signal coverage information for the whole genome. A Random Forest model was used to integrate those four categories of features. Meanwhile, we also built chromatin accessibility prediction solely based on TF binding features (TF model), HM features (HM model) or motif features (Motif Model). These models enabled us to quantify the power of each feature category in predicting chromatin accessibility.

### HMs and TFs served as major determinants the chromatin accessibility

**Fig. 2:**
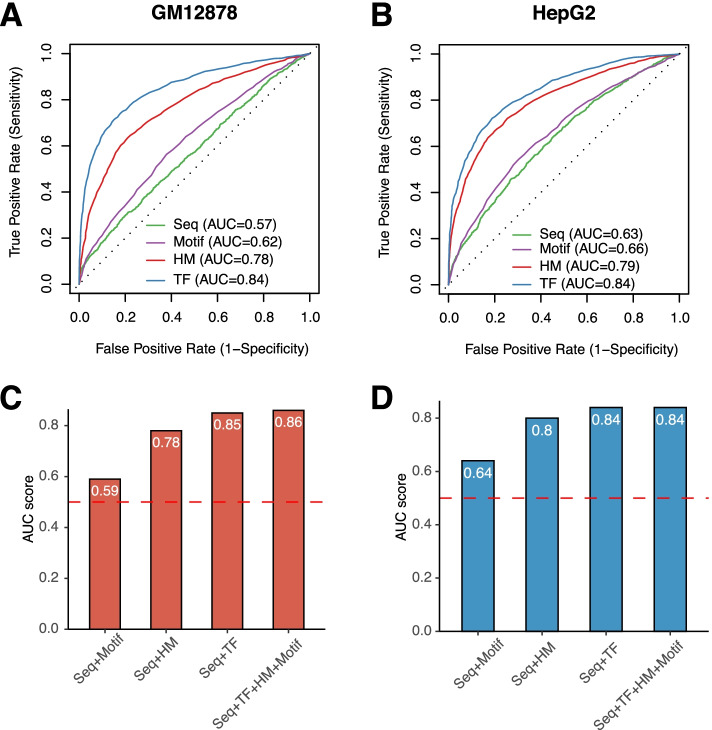
Histone and TF determines the chromosome accessibility. **A** and **B**. ROC curves of using DNA sequence, TF motif occurrence, HMs signal and TFs binding signal as features for chromatin accessibility prediction in GM12878 **(A)** and HepG2 **(B)** cell lines. **C** and **D**. Barplots showing the AUC of using combined DNA sequence + Motif, DNA sequence + HM, DNA sequence + TF and DNA sequence + Motif + HM + TF as features for chromatin accessibility prediction in GM12878 **(C)** and HepG2 **(D)** cell lines. All AUCs were calculated by taking the average AUC of 10-folds cross validation

We first quantified the AUC of each category of features in predicting chromatin accessibility using Random Forest models that incorporate just one specific set of features (TF, HM, TF motif, or DNA seq) (Fig. 2A-B). In GM12878 cell line, the highest AUC was obtained through the TF model (AUC = 0.84, Fig. 2A), followed by the AUC of the HM model (AUC = 0.78, Fig. 2A). The similar pattern was observed in HepG2 cell line, where TF model and HM model achieved an AUC of 0.84 and 0.79 respectively (Fig. 2B). This indicated that HM and TF had comparable power in predicting chromatin accessibility. On the contrary, a much weaker prediction power was observed for models based on sequence or motif features.

Then we examined the prediction performance of using different combinations of features. Our result indicated that the sequence and motif features did not further improve the prediction accuracy of the TF model and the HM model (Fig. 2C-D). In addition, when TF and HM features were combined, the resulting models achieved similar accuracy with the TF model (AUC = 0.84) and the HM model (AUC = 0.78), with the Seq + Motif+TF + HM model having an AUC of 0.86 in GM12878 (Fig. 2C). Similar results were observed in HepG2 (Fig. 2D). There results suggested that TF and HM features possessed redundant information in terms of chromatin accessibility prediction.

### Core histone-related features associated with chromatin accessibility

**Fig. 3:**
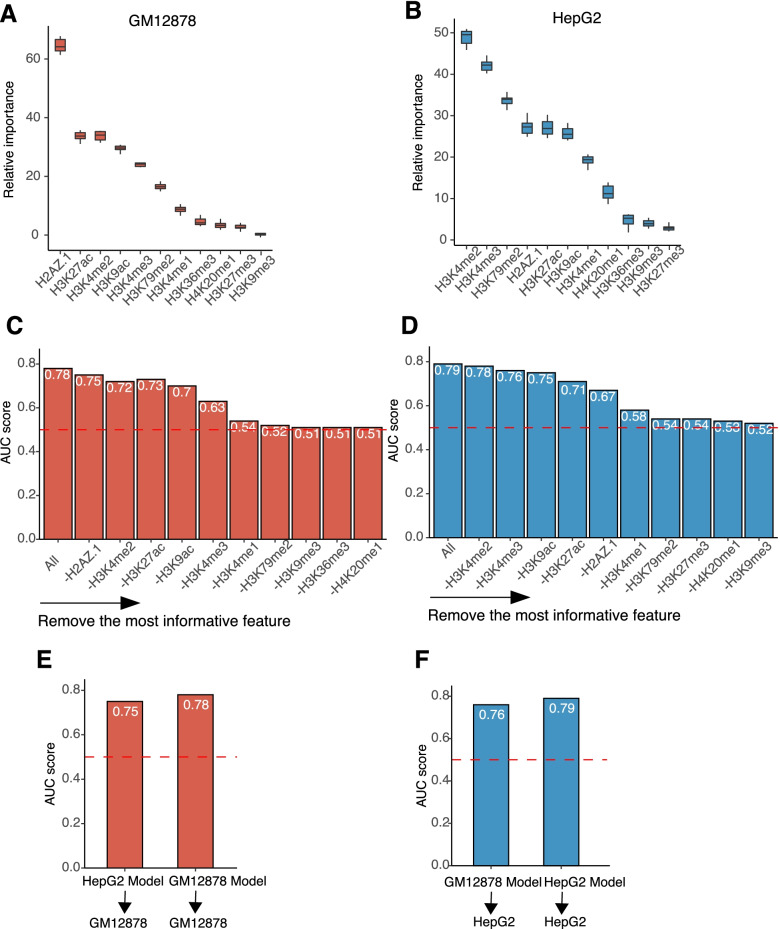
Histone models identify five core histones-related features with strong effects on chromosome accessibility. **A** and **B**. Boxplots indicating the relative importance of each histone-related features in chromatin accessibility prediction in GM12878 **(A)** and HepG2 **(B)** cell lines. **C** and **D**. Barplots showing the AUC change of taking out each HM for chromatin accessibility prediction in GM12878 **(C)** and HepG2 **(D)** cell lines. **E**. AUCs in (**C**) and (**D**) were calculated by taking the average AUC of 10-folds cross validation. Barplots showing the AUC of HepG2 model in GM12878 HM signals and GM12878 model in GM12878 HM signals for GM12878 chromatin accessibility prediction. **F**. Barplots showing the AUC of GM12878 model in HepG2 HM signals and HepG2 model in HepG2 HM signals for HepG2 chromatin accessibility prediction. AUCs of GM12878 model in GM12878 HM signals and HepG2 model in HepG2 HM signals were calculated by taking the average AUC of 10-folds cross validation. AUCs of GM12878 model in HepG2 HM signals and HepG2 model in GM12878 HM signals were calculated by applying the model to a different test dataset

HM functioned as a major individual determinant of the chromatin accessibility, so it is of interest to determine the contribution of each of the HM and to identify the important HMs for chromatin accessibility. Taking advantage of the Random Forest model, we derived the relative importance of each HM in the GM12878 and HepG2 cell lines respectively. Overall, the function of the HMs is very homogeneous in both cell lines. For example, H2AZ.1, a variant of histone H2A [20, 21], was found to be the most important HM in GM12878 cell line and the 4th important HM in HepG2 cell line (Fig. 3A-B). In contrast to H2AZ.1, the importance of H3K27me3 was consistently low in both cell lines. It ranked the second least important HM in GM12878 cell line and the least important HM in HepG2 cell line (Fig. 3A-B). More importantly, 5 histone-related features were found to have high relative importance in both two cell lines (Fig. 3A-B), suggesting that a core group of HMs was pivotal to the chromatin accessibility. To illustrate the essential role of those 5 histone-related features, we utilized a breakdown strategy to calculate the AUC after taking out the most important HMs. The prediction accuracy of the model kept consistently high in both cell lines (AUC > 0.7) when at least one of the 5 core group of HMs were still included in the model (Fig. 3C-D). However, after removing all H2AFZ, H3K4me2, H3K27ac, H3K9ac and H3K4me3 from the model, the prediction power of the model was heavily weakened (AUC = 0.63, GM12878; AUC = 0.67 HepG2, Fig. 3C-D).

Different HMs could have very similar function across different cell lines [11, 22]. For example, H3K4me3 is consistently associated with transcription start site (TSS) while H3K27ac is associated with enhancers [11]. We hypothesized that HMs also had conserved functions in regulating chromatin accessibility between different cell lines. To test this hypothesis, we first trained the HM model in the HepG2 cell line and achieved an AUC = 0.79 by 10-fold cross validation. Then we applied this validated HepG2 model to predict the chromatin accessibility in the GM12878 cell line using the HM signals of GM12878. Shown in Fig. 3E, the HepG2 model in the GM12878 cell line achieved similar prediction performance with GM12878 self-prediction performance and outperformed the random constructed model. Similar results can be shown with the same procedure of training the model in the GM12878 cell line dataset and testing on the HepG2 cell line dataset (Fig. 3F).

### Strong association of H2AZ.1 with chromatin accessibility

**Fig. 4:**
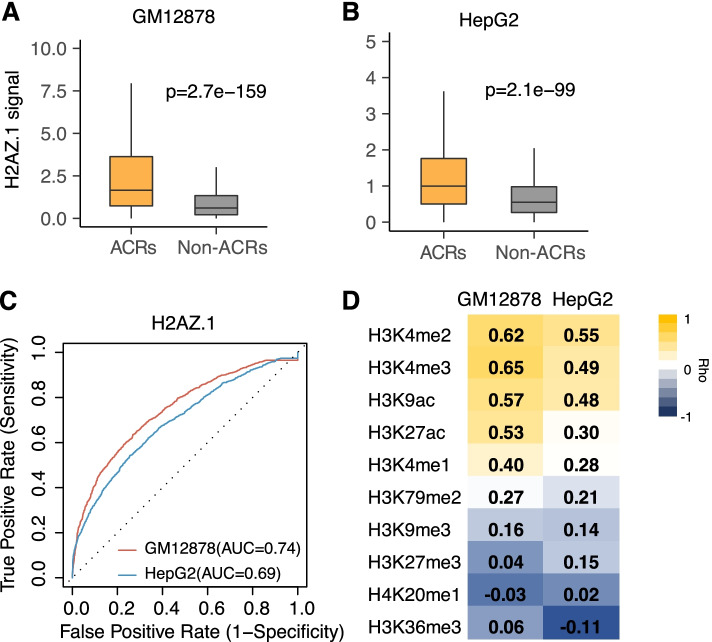
H2AZ.1 serves as major histone modifications in chromatin accessibility regulation. **A** and **B**. Boxplots indicating the H2AZ.1 signal difference in ACRs and non-ACRs. *P* value was calculated by Wilcoxon rank sum test. ACRs and non-ACRs were 2000 randomly chosen bins from the total ACRs and non-ACRs. **C.** ROC curves of using H2AZ.1 as feature for chromatin accessibility prediction in GM12878 and HepG2 cell lines. AUCs were calculated by taking the taking the average AUC of 10-folds cross validation. **D.** Heatmap showing the correlation between H2AZ.1 signals and other HM signals. The correlation coefficient was calculated by Spearman Correlation

In both the HepG2 and GM12878 cell lines, we found H2AZ.1 ranked at the top of the five core histone-related features regulating chromatin accessibility (Fig. 3A-D). H2AZ.1 is a variant of histone H2A and has found to be associated with active promoters and enhancers [20, 21]. Its function in regulating the global chromatin accessibility has not been well studied. We quantified the different H2AZ.1 signals in accessible chromatin regions (ACR) verses non-accessible chromatin regions (Non-ACR, Fig. 4A-B). The chromatin accessible regions had significantly higher H2AZ.1 signal compared to the inaccessible regions (*P* = 2.7e-159, GM12878; *P* = 2.1e-99, HepG2, Fig. 4A-B). And this significant higher pattern was consistent after further separating the accessible regions into proximal and distal regions (Suppl Fig. 2). Using H2AZ.1 signal as the predictor could stratify the accessible and inaccessible chromatin regions with high accuracy in both cell lines (AUC = 0.74, GM12878; AUC = 0.69, HepG2, Fig. 4C). These results revealed that H2AZ.1 impacted the chromatin accessibility in a global manner and was not limited to specific regulatory elements region. We further examined the potential synergized HMs with H2AZ.1 by examining the colocalization of H2AZ.1 signals with other HMs. The signals of H2AZ.1 colocalized with the rest of the core HMs in both cell lines with relative high correlation (Fig. 4D), indicating the synergistic effect among those 5 core HMs. In addition to those 5 core HMs, there was no strong negative association with other HMs’ signals (Fig. 4D).

### Cell line specific and non-specific TFs predicts chromatin accessibility

**Fig. 5:**
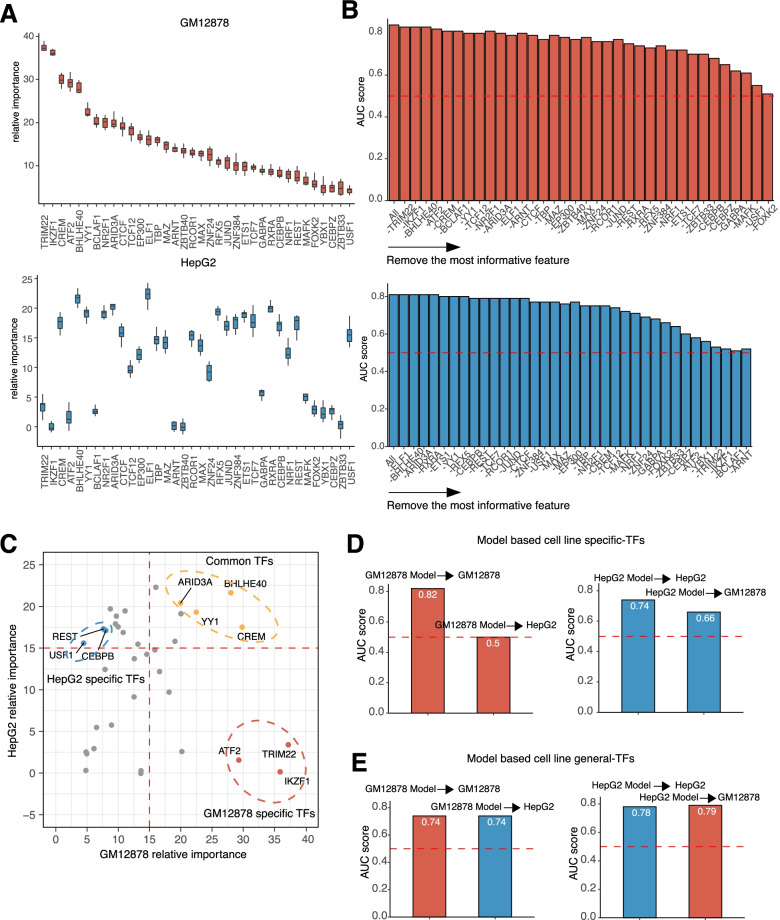
TF models identify cell line specific and shared specific TFs with strong effects on chromosome accessibility. **A**. Boxplots indicating the relative importance of each TFs binding signals in chromatin accessibility prediction in GM12878 (up panel) and HepG2 (bottom) cell lines. **B**. Barplots showing the AUC change of taking out each TF binding signal for chromatin accessibility prediction in GM12878 (up panel) and HepG2 (bottom) cell lines. **C**. Scatterplot showing the consistency of TFs relative importance between GM12878 and HepG2 cell lines. The circles indicated GM12878 specific TFs, HepG2 specific TFs and common TFs. **D**. Barplots showing the AUC of GM12878 specific TF model with GM12878 TFs binding signals for GM12878 chromatin accessibility prediction and GM12878 specific TF model with HepG2 TFs binding signals for HepG2 chromatin accessibility prediction (left panel). Barplots showing the AUC of HepG2 specific TF model with HepG2 TFs binding signals for HepG2 chromatin accessibility prediction and HepG2 specific TF model with GM12878 TFs binding signals for GM12878 chromatin accessibility prediction (right panel). **E**. Barplots showing the AUC of GM12878 common TF model with HepG2 TFs binding signals for HepG2 chromatin accessibility prediction and GM12878 specific TF model with HepG2 TFs binding signals for HepG2 chromatin accessibility prediction (left panel). Barplots showing the AUC of HepG2 common TF model with GM21878 TFs binding signals for GM12878 chromatin accessibility prediction and GM12878 common TF model with GM12878 TFs binding signals for GM12878 chromatin accessibility prediction (right panel). AUCs of GM12878 model in GM12878 TF signals and HepG2 model in HepG2 TF signals were calculated by taking the average AUC of 10-folds cross validation. AUCs of GM12878 model in HepG2 TF signals and HepG2 model in GM12878 TF signals were calculated by applying the model to a different test dataset

Compared with the HM model, the TF model had even better power in predicting chromatin accessibility (Fig. 2C-D). Unlike HMs, the roles of TFs are largely heterogeneous. Common TF could be shared among different cell types and directly bind to condensed chromatin to establish accessibility, while other TFs are recruited to the opened chromatin regions and act in a cell line specific manner. To investigate the roles of different TFs in opening chromatin, we ranked the relative importance of each TF in the GM12878 and HepG2 models. TRIM22, a T cell activation associated transcription factor [23], had the highest relative importance in the GM12878 cell line but not in HepG2, revealing its cell line specific role in regulating the chromatin accessibility (Fig. 5A). In addition to the cell line specific TF, we also identified YY1, a structure regulator of enhancer-promoter interactions [24–27], whose relative importance was high in both GM12878 and HepG2 cell lines (Fig. 5A). We further performed the similar breakdown analysis and sought to find a group of core TFs. Unlike the pattern of HMs breakdown analysis, the prediction accuracy dropped very smoothly. The model collapsed and presented a notable drop after removing most of the TFs in both cell lines (Fig. 5B). In addition, the TFs that caused the drop of the prediction were different in both cell lines, though some shared TFs were identified. For example, in GM12878 cell line, TRIM was removed in the very beginning in the model while it was removed at the very end in the HepG2 cell line. In contrast, YY1 was removed at the early stage in the breakdown analysis in both cell lines (Fig. 5B). These results revealed that both shared TFs and cell line specific TFs participate in regulating the chromatin accessibility.

In order to identify the cell line-specific and cell line shared TFs, we plotted the importance of different TFs in the GM12878 and HepG2 cell lines on a scatterplot (Fig. 5C). A weak correlation was observed between these two cell lines, supporting the hypothesis that TFs are largely heterogenous in regulating chromatin accessibility in different cell lines (Fig. 5C). However, there was a group of TFs (YY1, CREM, ARID3A and BHLHE40) that had high relative importance in both cell lines and therefore were defined as the common TFs (Fig. 5C). Additionally, we revealed two groups of TFs with high relative importance but limited to their own cell origins. For example, the TRIM22’s high relative importance was only observed in GM12878 cell line. Driven by this, we defined 3 GM12878 specific TFs, 3 HepG2 specific TFs and 4 cell line shared TFs to train the GM12878 specific, HepG2 specific and shared TF models. The predictive power of those TFs was validated through examining if those models could be transferred across the two cell lines. For GM12878 specific TF model, the prediction accuracy dropped from 0.82 to 0.5 when applying the model to HepG2 data (Fig. 5D). Similar finding could be observed when applying HepG2 specific TF model to the GM12878 data (Fig. 5D). In contrast, the shared TF models could be transferred between HepG2 and GM12878 cell lines (Fig. 5E). Either GM12878 or HepG2 common TF models could achieve comparable prediction accuracy in those two cell lines (Fig. 5E).

### HM and TF features are redundant for predicting chromatin accessibility

**Fig. 6:**
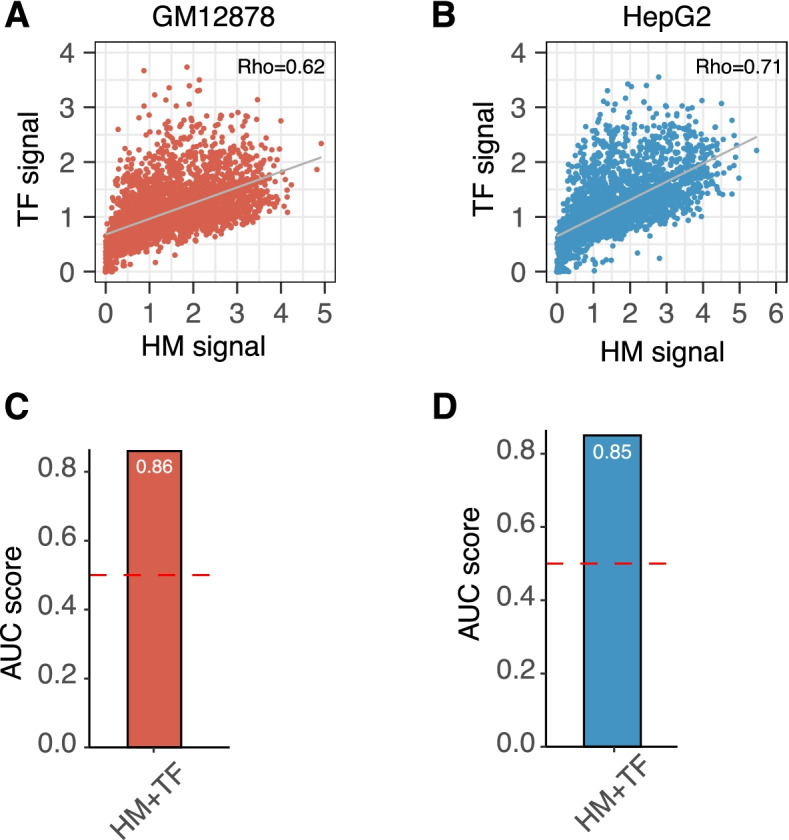
Redundancy between HM and TF features in determining chromosome accessibility. **A** and **B**. Scatterplot showing the correlation between histone modifications signals and TFs binding signals in GM12878 **(A)** and HepG2 **(B)** cell lines. The correlation coefficient was calculated by Spearman correlation. **C** and **D**. Barplots showing the AUC of combined HM with TF model in GM12878 **(C)** and HepG2 **(D)** cell lines. AUCs were calculated by taking the taking the average AUC of 10-folds cross validation

Epigenetic studies suggested that HMs play a central role in transcriptional regulation and revealed substantial overlaps between TFs binding and HMs [28]. In our analysis, both the histone model and the TF model achieved good performance in predicting chromatin accessibility (Figs. 2, 3 annd 4), leaving an open question whether HMs and TFs binding provide complementary or redundant information to each other in regulating the chromatin accessibility. We first examined the level of colocalization between HMs and TF binding signals through spearman correlation. On the genome of both the GM12878 and HepG2 cell lines, the average HM signals were highly correlated with the average TF binding signals (Rho = 0.62, GM12878; Rho = 0.71, HepG2, Fig. 6A-B). Therefore, we hypothesized that HM signals and TF binding signals provided redundant information in predicting chromatin accessibility. HMs only, TF binding only and TF-HM combined models were built and compared. In GM12878 cell line, the HMs only model achieved an AUC = 0.78 and the TFs only model achieved an AUC = 0.84. The AUC didn’t increase remarkably when integrating HMs with TFs (AUC = 0.86, Fig. 6C). The same finding was observed in HepG2 cell line (AUC = 0.79, HMs; AUC = 0.84, TFs; AUC = 0.85, HMs + TFs, Fig. 6D). In conclusion, TFs colocalized with HMs when regulating chromatin accessibility and provided redundant information to each other in chromatin accessibility prediction (Fig. 6).

### Accessible TSS proximal and distal regions presented distinctive epigenomic signals

**Fig. 7:**
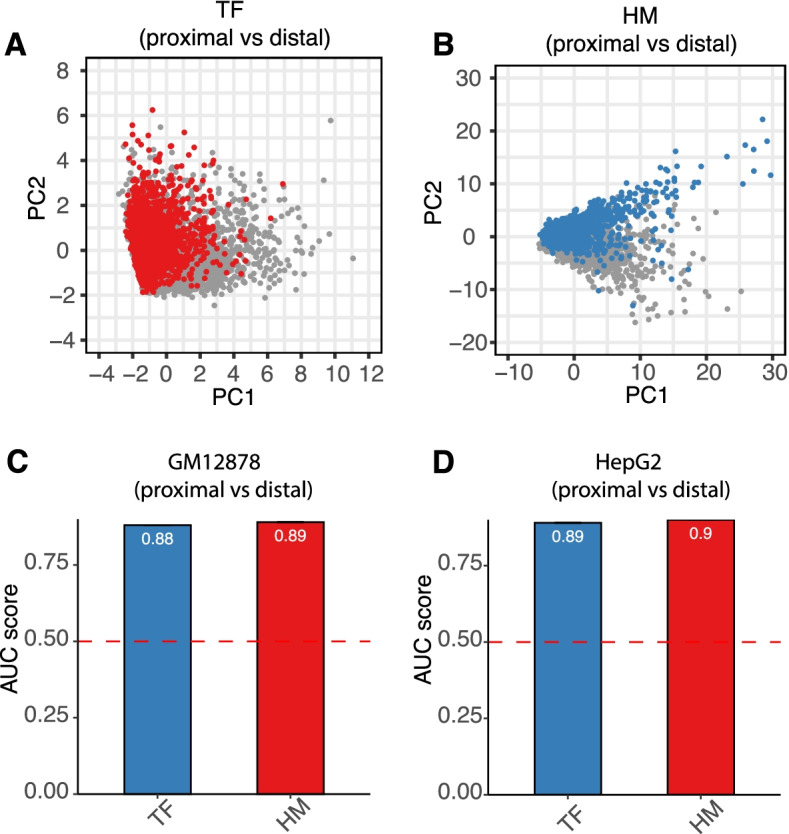
Accessible of TSS proximal and distal regions present distinctive epigenomic signals. **A** and **B**. PCA plot showing the separation of accessible TSS proximal region versus accessible distal region using TFs binding signals **(A)** and HMs **(B)**. Red and Blue dots indicated proximal regions. Grey dots indicated distal regions. **C** and **D**. Barplots showing the AUCs of using TFs binding signals and histone modifications signals for accessible proximal region versus distal region prediction in GM12878 **(C)** and HepG2 **(D)**. AUCs were calculated by taking the taking the average AUC of 10-folds cross validation

We have validated the efficacy of our integrative model in predicting the chromatin accessibility. The position of open chromatin varies, which could be further separated into TSS proximal and distal regions where both TFs and HMs were expected to present distinctive signals. Principal component analysis (PCA) showed a separation of TFs and HMs signals in the TSS proximal and distal region (Fig. 7A-B). This clear separation pattern introduced a chance of using our current model to predict the accessibility of a specific region. We thus applied our GM12878 TF and HM model defined in the beginning to predict the TSS proximal and distal regions’ accessibility. The TF model had AUC = 0.97 in predicting both the TSS proximal and distal regions’ accessibility (Suppl. Fig. 3A-B). Comparable performance was observed using the HM model (AUC = 0.92, proximal, AUC = 0.85, distal, Suppl. Fig. 3A-B). Similar results were observed In the HepG2 cell line (Suppl. Fig. 3C-D).

We further designed a detailed model for distinguishing the accessible TSS proximal regions with distal regions using HMs and TF binding signals. Both the TF and HM models obtained decent predicting power in both the GM12878 and HepG2 cell lines (AUC = 0.88, TF model in GM12878, AUC = 0.89, histone model in GM12878, AUC = 0.89, TF model in HepG2, AUC = 0.90, histone model in HepG2, Fig. 7C-D).

## Discussion

In this work, we here pioneered a two-layer quantitative integrative model for the prediction of chromatin accessibility. Two recent studies either used sequence feature or integrated sequence with TF expression or only integrated HMs with TFs signals for chromatin accessibility prediction [11, 14, 29]. Compared with the previous studies, our analysis had such advantages in the following aspects. First, we provided a generalized computational framework for epigenomic data integration. Through the development of the model, we integrated sequence features, which include sequence and TF motifs, with the epigenomic features, which include ChIP-seq profiles of HMs and TFs, for chromatin accessibility prediction. Second, we systematically evaluate the contribution of different groups of features in predicting chromatin accessibility and identify HMs and TFs severed as the major determinants. Third, we generated feature specific prediction model to explore the mechanism that HMs and TFs played in regulating chromatin accessibility.

Using sequence information for chromatin accessibility prediction or histone modification prediction has been well reported. In contrast to those previous results, our model presented a poor performance of using sequence information as the predictor (Fig. 1). The differences in prediction performance could be explained by the choice of positive and negative regions during model training. Most studies chose the random regions in the genome as the negative regions for prediction. In that case, instead of predicting chromatin accessible versus inaccessible regions or modified versus non-modified regions, the model was predicting chromatin accessible or modified regions versus random background. Our study selected the nearby regions of the accessible regions as the negative regions to minimize the sequence level variation and therefore provided an objective comparison.

As early in 1960, Allfrey et al. identified the acetylation of histones, leading to the hypothesis that acetylation is associated with transcription activity [30]. In the past decades, tremendous efforts have been made to illustrate the cellular functions of HMs [11]. Though the association between individual HM and TF with open chromatin has been studied for decades [11], the relative importance of each HM and TF in regulating chromatin accessibility was not addressed in a global manner. In our analysis, we first identified TFs and HMs served as the major determinants of chromatin accessibility (Fig. 2). Additionally, TFs and HMs cooperated together to regulate the chromatin accessibility (Fig. 6). It was well known that TFs interacts with HMs for regulating gene expression [31, 32]. Our analysis indicated this interaction happened beyond the TSS range by observing the high correlation structure in the genome wide level (Fig. 6). And it is not surprising to observe the prediction redundancy between HMs and TFs (Fig. 6). The mechanisms behind TFs and HMs regulation are complicated and multiple models have been proposed. For example, the TFs could interact with chromatin remodeler directly to create an accessible region whereas HMs are simply the subsequent readout. However, the power of our analysis was limited by the number of ChIP seq files provided in the ENCODE dataset. With more ChIP seq files released in the future, we could further investigate the relationship between TFs, chromatin remodeler and HMs in regulating chromatin accessibility.

We identified 4 important HMs and 1 histone variants which were all reported to be associated with active gene transcription or enhancers [1] (Fig. 3). Interestingly, the histone H2A variant H2AZ.1, which was found to be enriched in active promoters and enhancers [20, 21], was similar or more important in regulating chromatin accessibility compared to other well-known HMs (Fig. 4). This supports the theory that histone variants, though very similar in their major canonical histone counterparts, could have strong functional impacts [21, 33]. With more ChIP-seq files of histone variants being available, we could systematically compare the importance of histone variants with HMs in chromatin accessibility regulation. Interestingly, the importance of these 5 core HMs in regulating chromatin accessibility is shared between GM12878 and HepG2 cell lines. The prediction model that trained either in GM12878 or HepG2 cell line could be easily transferred across both cell lines, supporting the existence of a common histone code for chromatin accessibility [12] or at least those two cell lines shared the similar histone code.

Unlike the core HMs, different TFs regulate chromatin accessibility in different manners among cell lines. In our analysis, there was not a group of core TFs that were important for chromatin accessibility in both cell lines. We therefore developed a computational framework to identify common TFs and cell line specific TFs. Cell line specific TFs including IKZF1, TRIM22 and ATF2 only predict the chromatin accessibility in GM12878 cell line, though the ChIP-seq profiles of these cell line specific TFs were also measured in the other cell line. One of the reasons that make those TF important is they have a global gain of binding signals in the accessible regions compared with other TFs. We, therefore, examined the correlation between peak number and the relative importance of those TFs. A general positive correlation was observed in both GM12878 and HepG2 cell lines (Suppl. Fig. 5A-B).

GM12878 is a lymphoblastoid cell line from B lymphocyte. IKZF1 is an important regulator in B-cell precursor acute lymphoblastic leukemia [34]. ATF2’s function in regulating B cell lymphoma progression has been well illustrated in previous studies [35, 36]. Unlike IKZF1 and ATF2, TRIM22’s function was not well characterized in B cell associated cancer. TRIM22 is IFN-stimulated gene (ISG) upregulated upon IFN administration and its expression in B cell lymphoma has been reported [37]. Given its strong association with chromatin accessibility in GM12878 cell lines, further efforts are needed to illustrate its regulatory mechanism. HepG2 cell line is a liver cancer cell line where CEPB, REST and USF1 strongly contribute to chromatin accessibility. In specific, CEBB expression served as a prognostic marker in liver cancer and contribute to the progression of liver cancer. Though CEPB is identified as a tumor suppressor, its function has been controversially reported in liver cancer, yet more detailed investigation is needed [38, 39]. USF1 expression was found to be significantly higher in liver cancer tissue compared with normal liver cancer tissue [40]. As a member of c-Myc related family, USF1 could regulate numerous gene expression, which leads to liver cancer progression [41–43]. REST gene was marginally studied in liver cancer. There is only a handful of studies discussing its expression and alternative splicing status in cancer [44, 45]. Our results indicated its importance in maintaining chromatin accessibility. Soon, when the new ChIP-seq and ATAC-seq become available, we hope to further examine REST’s role in liver cancer progression.

Shared TFs between different cell lines also existed as the prediction model could be transferred due to their similar power in predicting chromatin accessibility across different cell lines (Fig. 5). Among those TFs, YY1 has been reported as the global regulator for promoter-enhancer interactions [24–27]. To our knowledge, the function of BHLHE40, ARID3A and CREM has not be well illustrated in terms of serving as common TF across different cell lines. To further examine their function, in the future, we could examine other cell lines which contained the ChIP-seq profiles of those 3 TFs for chromatin accessibility prediction.

Though ATAC-seq is the current golden standard for directly measuring chromatin accessibility, there were numerous chromatin accessibility data that were generated from other sequencing techniques [46–48]. Our model was not only able to predict chromatin accessibility measured by ATAC-seq, but also effectively predicted the accessibility that was measured by DNase- and FAIRE-seq data (Suppl. Fig. 4). With more data available, for example, the MNase-seq data [49], we could further validate the efficacy of our model. Notably, our model provided a novel way of re-utilizing the traditional ChIP-seq data. In ENCODE project, over 70 cell lines have the matched ChIP-seq data while only 11 cell lines provide ATAC-seq data. With the application of our framework, the chromatin accessibility of those cell lines could be inferred instead of performing additional ATAC-seq experiments.

## Conclusions

In summary, we developed a computational framework for chromatin accessibility modeling. Several important HMs and TFs were found as the major regulators of chromatin accessibility, providing novel mechanistic insights in chromatin accessibility regulation. The computational framework and the introduced integrative model in our study can be generally applied to for modeling different genomic and epigenomic data.

## Material and methods

### Datasets collection and processing

To construct integrative models to predict chromatin accessibility, we utilized the GM12878 and HepG2 cell lines, for which high quality chromatin accessibility, TF binding and histone modification have been generated by the Encyclopedia of DNA elements (ENCODE) project [50]. The chromatin accessibility data were obtained by using the ATAC-seq, DNase-seq and FAIRE-seq experiments and downloaded from the ENCODE database as bedgraph files. Specifically, for the GM12878 cell line, the accession IDs are ENCFF172DEA, ENCFF235KUD, and ENCFF001UYE for ATAC-seq, DNase-seq, and FAIRE-seq, respectively. For the HepG2 cell line, the accession IDs are ENCFF356TXH, ENCFF422EDI, and ENCFF001UYN for ATAC-seq, DNase-seq, and FAIRE-seq, respectively.

The ChIP-seq profiles of histone modifications and TFs were also downloaded from ENCODE as bigwig files. The GM12878 data contain 53 files with replicates for 11 different histone modifications; and 361 files with replicates for 97 different TFs. The HepG2 data contain 52 files with replicates for 11 different histone modifications; and 763 files with replicates for 221 different TFs. All files were mapped to the hg19 genome. The annotation of these ChIP-seq files is summarized in Suppl. Table 1. All the signals in the files were defined as log fold change of normalized reads compared to the input control.

### Identification of chromatin accessible and non-accessible regions

The ATAC-seq bedgraph files were used to define the chromatin accessible (positive) and non-accessible (negative) regions in the genome. Peaks in the ATAC-seq represent regions in the chromatin that are highly accessible to Tn5 transposase and correlate with transcription activity. The positive region was defined as the genomic regions covered by ATAC-seq peaks listed in the bedgraph files. The negative region was defined as the neighbor region at both sides of each ATAC-seq peak with the same lengths. Namely, the positive and negative regions had exactly the same bases length with number of positive-to-negative ratio of 1:2. In addition, the negative regions shared similar sequence features such as GC content with the corresponding positive region due to genomic proximity, making them ideal negative controls to the chromatin accessible regions. The sizes of different regions vary dramatically and therefore we divided each region (both positive and negative) into small bins of 100 bp in size, which is around the half-size of DNA covered by a single nucleosome [51]. The models were constructed at the bin level.

### Calculation of HM and TF binding signal profiles

For each bin, we calculated the average TF binding and HM signals based on the corresponding bigwig files, which provided the signals at nucleotide level, represented as the log ratios (the immunoprecipitation sample with respect to the control). The Deeptools software was used for calculating bin-specific HMs and TFs binding signals in both the positive and negative regions [52]. Specially, the *multiBigwigSummary* function was used with parameter “-bs 100” to calculate the average signal of each ChIP-seq file in a 100 bp window across the genome [52].

### Identification of TF motifs in genomic regions

DNA sequences for positive and negative regions were retrieved from the hg19 reference genome sequence downloaded from Ensembl website [53]. The occurrences of TF binding motifs were determined by using the FIMO program from the MEME Suite [54], which identified motifs by matching with positional weighted matrices (PWMs) of TFs. A total of 687 PWMs were used, which were downloaded from JASPAR and TRANSFAC database [55, 56]. FIMO was applied using a default parameter with a cut-off of *p*-value <1e-4.

### A two-layer integrative model for chromatin accessibility prediction

A two-layer classification model was constructed to integrate sequence, TF motifs, binding of TF, and HMs to predict chromatin accessibility at the bin level (100-bp bins). The inputs to this model are DNA sequence of each bin, the presence of 687 TF binding motifs (1 and 0 indicate presence and absence, respectively), TF binding signals (derived from ChIP-seq data), and HM signals (derived from ChIP-seq data). The output of this model is the accessibility of bins, with 1 and 0 indicating accessible and non-accessible, respectively.

In the first layer, a convolutional neural network (CNN) model was constructed to predict the baseline accessibility at the bin level solely based on the DNA sequences (100 bp) of bins since the CNN model has been widely used for modeling sequence based features [57]. The configuration of the CNN model is shown in Suppl. Fig. 1. Each of our convolutional layer contained 320, 480, and 640 hidden neurons, and the output of each convolutional layer was activated by the ReLU function before propagating to the next maxpooling or convolutional layers. We implemented a fully connected layer with ReLU activation on top of the three convolutional layers, which was further propagated to the output sigmoid layer to compute the probability of a given input sequence having an open chromatin state. The raw DNA sequences were encoded using “One-hot” encoding scheme as the input to the model, where each base pair in the sequence is mapped to the four possible nucleotides, with the corresponding nucleotide set as 1 and the other three set as 0. The output of this model is a score ranging between [0,1], indicating the probability of the bin to be accessible at the baseline level (i.e., fully based on its 100-bp DNA sequence). The implement of CNN model was finished using the *keras* package in R. In the second layer, the baseline accessibility score was combined with TF motifs, binding of TF and HMs to predict the two-class bin labels (accessible vs. non-accessible bins). These features are integrated by using the Random Forest classification model. Many TFs and HMs have multiple ChIP-seq replicates and we chose the one with the largest sequencing depths as the predicting feature so that for each TF/HM a single feature was used for easy model interpretation. We also evaluated different methods of choosing replicates. In specific, we applied three methods: 1) chose representative replicate with the largest sequencing depths; 2) used the average binding signal of all replicates as the representative replicate; 3) randomly chose one replicate as the representative replicate. The performance of those methods was consistent, indicating the high reproducibility of those replicates (Suppl Fig. 1B). We, therefore, stuck with the original method by choosing the representative replicate with the largest sequencing depth. To train the model, we used the ATAC-seq data for GM12878 and HepG2 cell lines. The positive (accessible) and negative (non-accessible) bins are defined as described in section *“Identification of chromatin accessible and non-accessible regions”*. The model was evaluated by 10-folds cross-validation using AUC (Area under ROC curve) score as the metric, which summarizes the specificity and sensitivity of classification models at different thresholds. Specifically, the whole data were divided into 10 subsets of equal sizes. Each time, 9 subsets were selected and combined as the training data and the remaining subset was used as the test data. The model was trained in the training data and then applied to the test data to predict the probability of bins to be accessible. This procedure was iterated until every bin had been in a test data and predicted exactly once. The predicted values are then used to calculate the AUC to evaluate the model performance. The 10-folds cross-validation training was implemented using a Random Forest model in R using the *randomForest* package.

### TF and HM prediction model and model transfer

To construct the feature-specific model for quantifying the contribution of DNA sequence, TF motifs, TF binding signals, and HM modification signals, we separated the input data from the original two-layer model into different subsets based on the characteristics of the predictors. Then a Random Forest model with 10-folds cross-validation was constructed to examine the prediction accuracy of each feature group. We further performed model transfer analysis to examine investigate if TF and HM models could be shared between different cell lines. In specific, the TF and HM models were directly trained in one cell line using the Random Forest model with 10-folds cross-validation. The trained TF and HM models then were applied in the other cell line for predicting its corresponding chromatin accessibility. For example, using GM12878 cell line trained TF and HM models for predicting chromatin accessibility in HepG2 cell line.

### TF and HM prediction model optimization and stability analysis

To optimize the second layer RF model, we applied a backward feature selection method. Initiated from the full model including all features, the feature with the least relative importance was removed iteratively. In each iteration, the performance of the model (AUC) was calculated based on 10-folds cross-validation. The optimized model was determined by visualizing the “number of features” versus AUC plot. A similar procedure was used to determine the minimum number of features required to achieve fairly high prediction accuracy. Features were removed from the full model one by one, but each time the one with the largest relative importance was removed until a sudden decrease of cross-validation AUC was observed.

### Model for classifying TSS-proximal versus distal accessible chromatin regions

It is known that both TF binding and HM signals have spatial patterns. Some HMs are enhancer associated while others are promoter associated. Therefore, we constructed models to classify transcription start site (TSS) proximal versus distal accessible chromatin regions. ChIPseeker package was used to categorize the accessible regions [58] into TSS-proximal and TSS-distal regions. The former was defined as accessible chromatin regions near the +/− 3000 bp of TSS, while the latter as the regions having no overlap with TSS proximal regions or exon regions. Using the TF binding and HM signals as the predictors, a Random Forest model with 10-folds cross-validation was constructed to classify the two categories of accessible regions.

## Supplementary Information

**Additional file 1: Suppl. Fig. 1.** The schematic overview of the CNN model and validation of replicates chosen.

**Additional file 2: Suppl. Fig. 2.** H2AZ.1 presents distinctive signals in proximal and distal regions. (A) and (B). Boxplots indicating the H2AZ.1 signal difference in Proximal ACRs and non-ACRs in GM12878 (A) and HepG2 (B) cell lines. (C) and (D). Boxplots indicating the H2AZ.1 signal difference in Distal ACRs and non-ACRs in GM12878 (C) and HepG2 (D) cell lines. *P* value was calculated by Wilcoxon rank sum test. ACRs and non-ACRs were 2000 randomly chosen bins from the total ACRs and non-ACRs.

**Additional file 3: Suppl. Fig. 3.** TFs and HMs are associated with accessible TSS proximal and distal regions. (A) and (B). Barplots showing the AUC of using TF model and HM model for accessible TSS proximal region (A) and distal region (B) prediction in GM12878 cell line. (C) and (D). Barplots showing the AUC of using TF model and HM model for accessible TSS proximal region (C) and distal region (D) prediction in HepG2 cell line. AUCs were calculated by applying the model to a different test dataset.

**Additional file 4: Suppl. Fig. 4.** TF and HM models predict chromatin accessibility measured by DNase-seq or FAIRE-seq. (A) and (B). Barplots showing the AUC of using TF model and HM model for DNase-seq measured chromatin accessibility (A) and FAIRE-seq measured chromatin accessibility (B) prediction in GM12878 cell line. AUCs were calculated by applying the model to a different test dataset.

**Additional file 5: Suppl. Fig. 5.** Correlation between peak number and relative importance. Scatterplot indicated the correlation between peak number and relative importance in A) GM12878 and B) HepG2 cell lines.

**Additional file 6: Suppl. Table 1.** Dataset summary. For each HM and TF, its ENCODE accession number, experimental targets and the cell line origin are provided.

## Data Availability

Not applicable.

## Abbreviations

HM: Histone modification
TF: Transcription factor
TSS: Transcription start site
ACR: Accessible chromatin region
Non-ACR: Non-accessible chromatin region
CNN: Convolutional Neural Network
ENCODE: The Encyclopedia of DNA elements
