# Supplementary figures and images for: Computational modeling of chromatin accessibility identified important epigenomic regulators
Source: BMC Genomics. 2022 Jan 8;23:19. doi: 10.1186/s12864-021-08234-5 (PMC8742372; doi:10.1186/s12864-021-08234-5)

**A**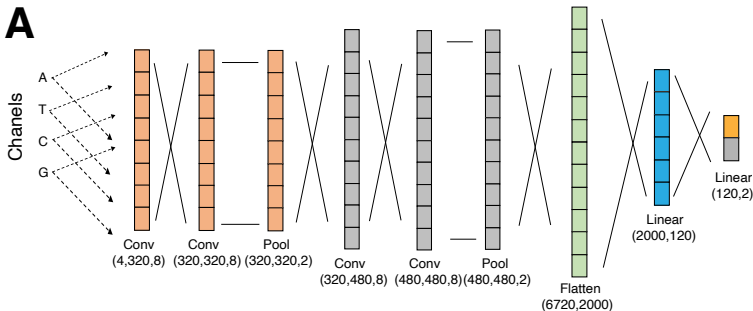**B****GM12878**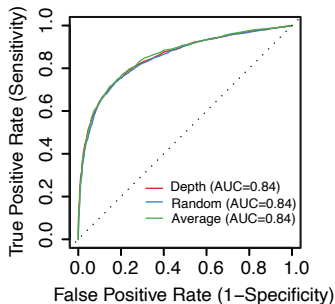**C****HepG2**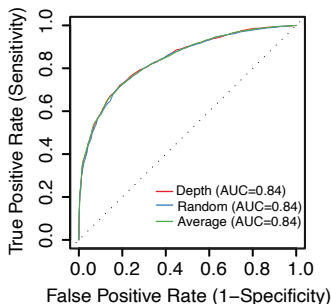

Supplement: Supplementary file 1 — Additional file 1: Suppl. Fig. 1. The schematic overview of the CNN model and validation of replicates chosen. [file 12864_2021_8234_MOESM1_ESM.pdf]

**A**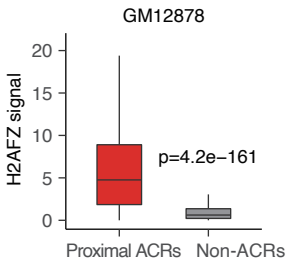**B**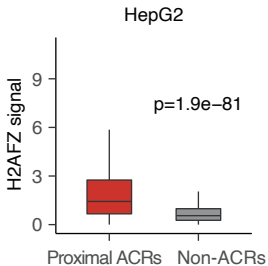**C**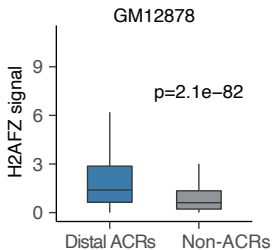**D**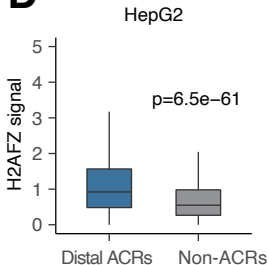

Supplement: Supplementary file 2 — Additional file 2: Suppl. Fig. 2. H2AZ.1 presents distinctive signals in proximal and distal regions. (A) and (B). Boxplots indicating the H2AZ.1 signal difference in Proximal ACRs and non-ACRs in GM12878 (A) and HepG2 (B) cell lines. (C) and (D). Boxplots indicating the H2AZ.1 signal difference in Distal ACRs and non-ACRs in GM12878 (C) and HepG2 (D) cell lines. P value was calculated by Wilcoxon rank sum test. ACRs and non-ACRs were 2000 randomly chosen bins from the total ACRs and non-ACRs. [file 12864_2021_8234_MOESM2_ESM.pdf]

**A**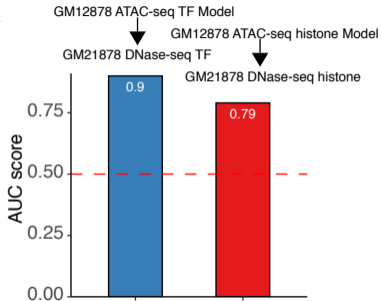**B**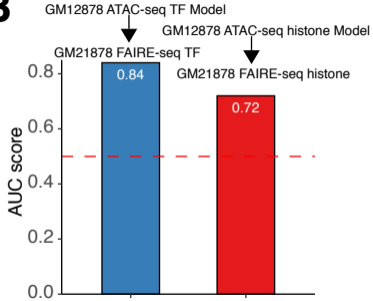

Supplement: Supplementary file 4 — Additional file 4: Suppl. Fig. 4. TF and HM models predict chromatin accessibility measured by DNase-seq or FAIRE-seq. (A) and (B). Barplots showing the AUC of using TF model and HM model for DNase-seq measured chromatin accessibility (A) and FAIRE-seq measured chromatin accessibility (B) prediction in GM12878 cell line. AUCs were calculated by applying the model to a different test dataset. [file 12864_2021_8234_MOESM4_ESM.pdf]

**A**

GM12878

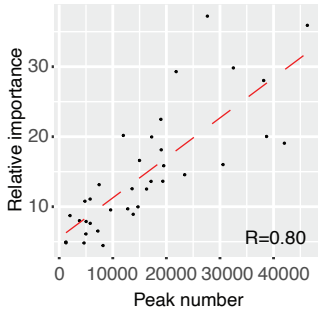**B**

HepG2

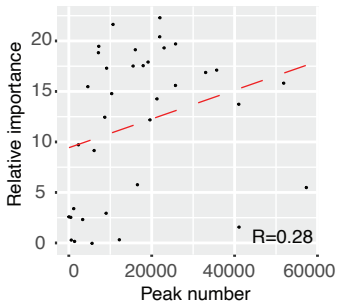

Supplement: Supplementary file 5 — Additional file 5: Suppl. Fig. 5. Correlation between peak number and relative importance. Scatterplot indicated the correlation between peak number and relative importance in A) GM12878 and B) HepG2 cell lines. [file 12864_2021_8234_MOESM5_ESM.pdf]
