# Supplementary material for: Computational modeling of chromatin accessibility identified important epigenomic regulators
Source: BMC Genomics. 2022 Jan 8;23:19. doi: 10.1186/s12864-021-08234-5 (PMC8742372; doi:10.1186/s12864-021-08234-5)
Supplement: Supplementary file 3 — Additional file 3: Suppl. Fig. 3. TFs and HMs are associated with accessible TSS proximal and distal regions. (A) and (B). Barplots showing the AUC of using TF model and HM model for accessible TSS proximal region (A) and distal region (B) prediction in GM12878 cell line. (C) and (D). Barplots showing the AUC of using TF model and HM model for accessible TSS proximal region (C) and distal region (D) prediction in HepG2 cell line. AUCs were calculated by applying the model to a different test dataset. [file 12864_2021_8234_MOESM3_ESM.pdf]

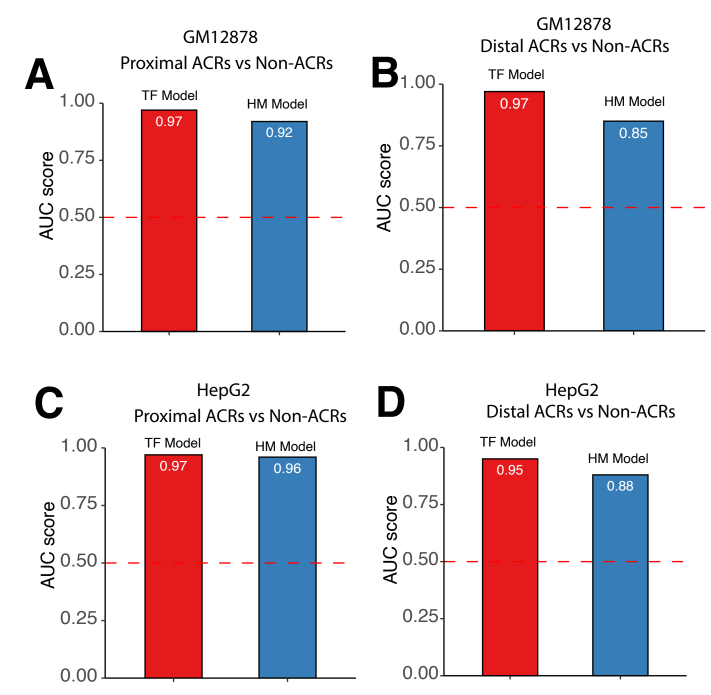

A
GM12878
Proximal ACRs vs Non-ACRs
AUC score
1.00
0.75
0.50
0.25
0.00
TF Model
0.97
HM Model
0.92
B
GM12878
Distal ACRs vs Non-ACRs
AUC score
1.00
0.75
0.50
0.25
0.00
TF Model
0.97
HM Model
0.85
C
HepG2
Proximal ACRs vs Non-ACRs
AUC score
1.00
0.75
0.50
0.25
0.00
TF Model
0.97
HM Model
0.96
D
HepG2
Distal ACRs vs Non-ACRs
AUC score
1.00
0.75
0.50
0.25
0.00
TF Model
0.95
HM Model
0.88
